# Pathways Involved in the Synergistic Activation of Macrophages by Lipoteichoic Acid and Hemoglobin

**DOI:** 10.1371/journal.pone.0047333

**Published:** 2012-10-10

**Authors:** Kathleen H. Cox, Michelle E. Cox, Virginia Woo-Rasberry, David L. Hasty

**Affiliations:** Department of Anatomy and Neurobiology, The University of Tennessee Health Science Center, Memphis, Tennessee, United States of America; Chang Gung University, Taiwan

## Abstract

Lipoteichoic acid (LTA) is a Gram-positive cell surface molecule that is found in both a cell-bound form and cell-free form in the host during an infection. Hemoglobin (Hb) can synergize with LTA, a TLR2 ligand, to potently activate macrophage innate immune responses in a TLR2- and TLR4-dependent way. At low levels of LTA, the presence of Hb can result in a 200-fold increase in the secretion of IL-6 following macrophage activation. Six hours after activation, the macrophage genes that are most highly up-regulated by LTA plus Hb activation compared to LTA alone are cytokines, chemokines, receptors and interferon-regulated genes. Several of these genes exhibit a unique TLR4-dependent increase in mRNA levels that continued to rise more than eight hours after stimulation. This prolonged increase in mRNA levels could be the result of an extended period of NF-κB nuclear localization and the concurrent absence of the NF-κB inhibitor, IκBα, after stimulation with LTA plus Hb. Dynasore inhibition experiments indicate that an endocytosis-dependent pathway is required for the TLR4-dependent up-regulation of IL-6 secretion following activation with LTA plus Hb. In addition, interferon-β mRNA is present after activation with LTA plus Hb, suggesting that the TRIF/TRAM-dependent pathway may be involved. Hb alone can elicit the TLR4-dependent secretion of TNF-α from macrophages, so it may be the TLR4 ligand. Hb also led to secretion of high mobility group box 1 protein (HMGB1), which synergized with LTA to increase secretion of IL-6. The activation of both the TLR2 and TLR4 pathways by LTA plus Hb leads to an enhanced innate immune response.

## Introduction

The mammalian innate immune system is responsible for the initial response to invading pathogens of various types. Their presence is detected by the reaction of conserved pathogen-associated molecular patterns (PAMPs) with pattern recognition receptors (PRRs) that are present in a variety of cell types in all multicellular organisms. The macrophage expresses several of these PRRs on its surface and within internal organelles, and is often the first cell type to detect and respond to PAMPs. The activation of PRRs initiates signal transduction pathways that determine the type and duration of the host inflammatory response [1]–[3].

One of the best-characterized families of PRRs is the Toll-like receptor (TLR) family. These receptors, as homo- or heterodimers, can be activated by bacterial, viral or fungal PAMPs. Response to lipopolysaccharide (LPS), one of the major Gram-negative PAMPs, is dependent upon TLR4 [4] and several co-receptors, such as CD14 [5], MD2 [6], [7] and LPS-binding protein [8], [9]. Responses to some cell surface components of Gram-positive bacteria, such as lipoproteins (including synthetic lipopeptides such as Pam2CSK4 and Pam3CSK4) and lipoteichoic acids (LTAs), require TLR2/TLR1 or TLR2/TLR6 heterodimers [10] and, in some cases, CD36 [11]. These TLRs are expressed on the plasma membrane and trigger unique signal transduction pathways both from the surface of macrophages, as well as from endocytic vesicles within the cell [12]–[14].

LTAs are abundant surface components of virtually all Gram-positive bacteria. The structures of LTA from different bacterial species vary somewhat, but all consist of a glycolipid moiety anchored in the cell membrane and a charged polyglycerolphosphate moiety extending into the cell wall [15]–[17]. The negative charge of the glycerolphosphate polymer is neutralized to different degrees by various substituents, such as D-alanine. In addition to a cell-bound form, LTA is also released into the surrounding milieu during growth in broth or during infections [18], [19]. Release of LTA can either be increased or decreased by treating infections with cell wall-active or protein synthesis-inhibiting antibiotics, respectively [20], [21]. LTAs are capable of triggering an immune response by interaction with TLR2/TLR6 heterodimers that are present on various mammalian cell types, including macrophages. The fatty acid groups of triacylated lipopeptides are the ligand for TLR2/TLR1 heterodimers [22], and the fatty acid groups of diacylated lipopeptides and LTA are ligands for TLR2/TLR6 heterodimers [23], [24].

Hemoglobin (Hb) greatly potentiates the secretion of cytokines and chemokines by LTA-activated mouse macrophages and human blood cells [25]. Unlike activation with LTA alone which only requires TLR2, this synergistic activation requires both TLR2 and TLR4 [26]. This interaction between LTA, Hb and macrophages could be quite significant in microenvironments in the body where damaged tissue comes in contact with bacterial components such as arterial plaques [27] or periodontal tissues [28]. In addition, a number of Gram-positive pathogens produce hemolysins that could also increase concentrations of free Hb in the surrounding area [29]. The present study was undertaken to further our understanding of the pathways involved in the synergistic effects that LTA and Hb have on macrophage activation.

## Materials and Methods

### LTA Purification

LTA was prepared from the M1 *S. pyogenes* strain, 8004, a clinical strain obtained from a patient with a severe invasive *S. pyogenes* infection [30] using the procedures of Morath et al. [31]. Briefly, the bacteria were grown overnight without shaking at 37°C in Todd Hewett broth with 1% yeast extract, collected by centrifugation and resuspended in 0.1 M sodium citrate buffer (pH 4.7). After mechanical disruption (Bead-Beater, Biospec Products), the bacteria were shaken for 30 min with an equal volume of *n*-butanol. The aqueous phase was dialyzed extensively against distilled water, lyophilized, and resuspended in 0.1 M ammonium acetate buffer (pH 4.7) containing 15% 1-propanol. The materials were fractionated by hydrophobic interaction chromatography on an octyl-Sepharose column, eluting with a linear 15–80% propanol gradient. Phosphorus-containing fractions were pooled, dialyzed extensively against distilled water, lyophilized, and resuspended in pyrogen-free water. LTA concentrations were determined by phosphorus analysis using the procedure of Gao et al. [32]. The level of endotoxin contamination was assayed using the QCL-1000 quantitative chromogenic Limulus amoebocyte lysate assay (Cambrex) (25). We used LTA from two different isolations in these experiments. The LAL reactivities were 1 and 5 EU/mg LTA possibly representing LPS levels of approximately 0.1 and 0.5 pg/µg LTA, respectively. In all of our experiments, we used 1–2 µg/ml of LTA and 5 µg/ml of polymyxin B (Sigma) as an LPS inhibitor. This concentration of polymyxin B completely inhibited activation of macrophages by >100 ng/ml of LPS (data not shown). We define complete inhibition as undetectable levels of secreted IL-6 or TNF-α, as well as levels of mRNA equal to control for all of the cytokine or chemokine genes examined. Thus, the level of LPS required to generate a response in our system would be at least 200,000-fold greater than the maximum levels found in our LTA preparations.

### Cell Culture

Most experiments utilized HeNC2 and GG2EE cells, immortalized mouse bone marrow macrophage cell lines derived from the C3H/HeN (TLR4^+^/_+_) and C3H/HeJ (TLR4^d^/_d_) mouse strains, respectively [33], obtained from Dr. Fabio Re. The cells were maintained in RPMI 1640 supplemented with 10% FBS and penicillin and streptomycin. In some experiments, we used bone marrow-derived macrophage cells isolated from C3H/HeOuJ (Tlr4^+^/_+_) or C3H/HeJ (Tlr4^d^/_d_) mice and differentiated according to the procedure of Zhang et al. [34]. Mice were used in strict accordance with the recommendations in the Guide for the Care and Use of Laboratory Animals of the National Institutes of Health. The protocol was approved by the Institutional Animal Care and Use Committee of the University of Tennessee Health Science Center (Animal Welfare Assurance Number: A3325-01). Cells were seeded at 2.5×10^5^ cells/well in 24-well plates in serum-free macrophage medium (Invitrogen) and allowed to recover for 2 hours before being stimulated, as indicated in the figure legends. Stock solutions of hemin (Sigma) were dissolved in 0.1 N NaOH and diluted into medium immediately before use. Stock solutions of Hb (Sigma) were also diluted with medium and both were assayed by LAL for endotoxin contamination. All the Hb preparations used for these experiments had LAL reactivities of 4 EU/mg Hb or less, and the heme preparation had a LAL reactivity of 8 EU/mg heme. All experiments contained 5 µg/ml polymixin B to inhibit any residual LPS. As stated above, this level of polymixin B completely inhibited activation of macrophages by >100 ng/ml LPS. Thus, it is highly unlikely that LPS contributed to any of the results presented here. Supernatants were isolated, spun at 2000 rpm for 5 min to remove any cells or cellular debris and frozen until cytokine determination. The concentrations of IL-6 and TNF-α were determined using ELISAs (eBioscience).

### Microarrays

HeNC2 cells were stimulated with LTA (2 µg/ml), Hb (50 µg/ml), LTA plus Hb (2 µg/ml and 50 µg/ml, respectively), or maintained in medium alone for 6 hours. RNA was isolated from three biological replicates of each condition. 200 ng of total RNA with a RIN score (RNA Integrity score) greater than 7 was used to generate cDNA and cRNA using Illumina TotalPrep RNA amplification kits (Ambion). From each of 12 samples, 1.5 µg of cRNA was hybridized overnight to the Mouse-6 v1B BeadChip in a multiple step procedure, according to the manufacturer’s instructions. The chips were washed, dried, and scanned on a BeadArray Reader (Illumina) and the raw data were generated using BeadStudio 2.3.41 software (Illumina).

### Microarray Analysis

The raw data were normalized with the quantile method using BeadStudio software. Differentially expressed genes were identified using the following criteria: 1) a 1.5-fold or greater change between experimental and control, 2) a mean GCOS-generated detection value >0.05 for at least the experimental value, and 3) a Welch t test p value of <0.05 for significance between the experimental and control.

### Quantitative Real-Time Reverse-Transcription Polymerase Chain Reaction (qRT-PCR)

Total RNA was isolated using Trizol (Invitrogen) and used as template to synthesize first strand cDNA. 1 µg RNA was reverse-transcribed using Transcriptor reverse transcriptase (Roche), random hexamer primers and anchored-oligo(dT)_18_ primers. Subsequent qRT-PCR analysis was carried out using a LightCycler 480 System (Roche) using primers specific for each gene (Supplemental Table 1) and probes from the Universal Probe Library system (Roche). The expression of both target genes and the control gene (*hgprt*) were quantified based on their threshold cycle values and the target genes were normalized using the control gene value. All values are expressed as the ratio of experimental conditions to the medium control.

**Table 1:** Genes up-regulated at least 2.5-fold when macrophages were activated by LTA plus Hb compared to macrophages activated by LTA alone.

Gene Symbol	Gene Name	Fold Enrichment	P-Value
**Chemokines and Cytokines**			
CCL2/MCP1	Chemokine (C-C Motif) Ligand 2	2.6	5.85E-04
CCL5/RANTES	Chemokine (C-C Motif) Ligand 5	12.5	3.69E-06
CCL7/MCP3	Chemokine (C-C Motif) Ligand 7	2.9	1.95E-05
CXCL10/IP-10	Chemokine (C-X-C Motif) Ligand 10	4.8	9.71E-06
CXCL2/MIP2	Chemokine (C-X-C Motif) Ligand 2	3.1	5.23E-06
IL1α	Interleukin 1α	3.0	1.84E-04
IL1β	Interleukin 1β	3.5	6.10E-05
IL27	Interleukin 27	3.4	4.35E-06
IL6	Interleukin 6	4.0	1.58E-03
**Receptors**			
CCRL2	Chemokine (C-C Motif) Receptor-like 2	2.9	1.18E-04
CD40	CD 40, TNF Receptor Superfamily Member 5	10.2	7.87E-05
**Interferon-Induced**			
GBP1	Guanylate Binding Protein 1	4.7	6.90E-05
GBP2	Guanylate Binding Protein 2	2.7	1.04E-05
GBP3	Guanylate Binding Protein 3	3.3	6.75E-05
IFIT2	Interferon-Induced Protein with Tetratricopeptide Repeats 2	2.9	1.27E-04
IFIT3	Interferon-Induced Protein with Tetratricopeptide Repeats 3	2.9	2.37E-05
MX2	Myxovirus Resistance 2	3.3	2.89E-05
**Enzymes**			
CMPK2/TYKI	Cytidine Monophosphate (UMP-CMP) Kinase 2, Mitochondria	3.5	3.11E-04
GSTT4	Glutathione S-Transferase Theta	2.6	4.53E-05
MMP13	Matrix Metallopeptidase 13	2.8	7.66E-05
OASL1	2′-5′-Oligoadenylate Synthetase-like	2.7	1.16E-04
TREX1	Three Prime Repair Exonuclease 1	2.8	2.03E-05
USP18	Ubiquitin Specific Peptidase 18	2.7	1.16E-05
**Trafficking**			
LCN2	Lipocalin	2.6	3.39E-04
MARCKS	Myristoylated Alanine-Rich Protein Kinase C Substrate	2.6	2.98E-06
**Other**			
RSAD2	Radical S-Adenosyl Methionine Domain Containing 2	5.6	1.21E-05
TRAF1	TNF Receptor-Associated Factor 1	2.5	7.01E-05

### Western Blots

HeNC2 cells were seeded at 5×10^5^ cells/well in 12-well plates in serum-free macrophage medium (Invitrogen) and allowed to recover for 2 hours before being stimulated, as indicated in the text and figure legends. For experiments assaying high mobility group box 1 protein (HMGB1), supernatants were collected, centrifuged to remove cells and cellular debris, and 10 µl samples were fractionated by SDS-PAGE and transferred to nitrocellulose for Western blot analysis. For experiments assaying IκBα, cells were lysed with M-PER Mammalian protein extraction reagent (Thermo Scientific) supplemented with Halt protease, phosphatase inhibitor cocktail and 0.5 mM EDTA (Thermo Scientific). Protein concentrations were quantified using the BCA protein reagent (Pierce) and 15 µg of total cellular lysate was analyzed by SDS-PAGE followed by Western blotting. Proteins were transferred to nitrocellulose at 4°C for 1 hour. Membranes were blocked in either 5% BSA or 5% non-fat milk in Tris-buffered saline solution with 0.05% Tween 20 (TBS-T) and incubated overnight at 4°C with antibodies against IκBα (1∶1000, Cell Signaling) or HMGB1 (1∶1000, Sigma) diluted in blocking solution. After washing, blots were incubated with goat anti-rabbit IgG peroxidase-conjugate (1∶10,000 Cappel) diluted in blocking solution and immunoreactive species were detected by ECL Western blotting detection reagent (Pierce).

### Immunolocalization Analysis

HeNC2 cells were seeded at 1×10^6^ cells/well in 6-well plates in serum-free macrophage medium (Invitrogen) on coverslips and allowed to recover for 2 hours before being stimulated, as indicated in the text and figure legends. Cells were then washed in phosphate-buffered saline (PBS), fixed with 4% paraformaldehyde in PBS, permeabilized by incubation in 0.25% saponin in PBS for 15 minute at room temperature and blocked for 1 hour in 10% goat serum in PBS. Following blocking, the cells were incubated with rabbit monoclonal anti-NF-κB p65 (D14E12, Cell Signaling) at 1∶50 in blocking solution overnight at 4°C. After washing with PBS, the cells were incubated with goat anti-rabbit Alexa Fluor-488 at 1∶1000 in blocking solution at room temperature for 1 hour. The cells were then washed in PBS and the localization of fluorescently labeled NF-κB (green) was visualized using a Zeiss LSM 510 laser scanning microscope. Nuclei were visualized with SYTOX orange (blue).

## Results

### Synergism between Hb and TLR2 Ligands

**Figure 1:**
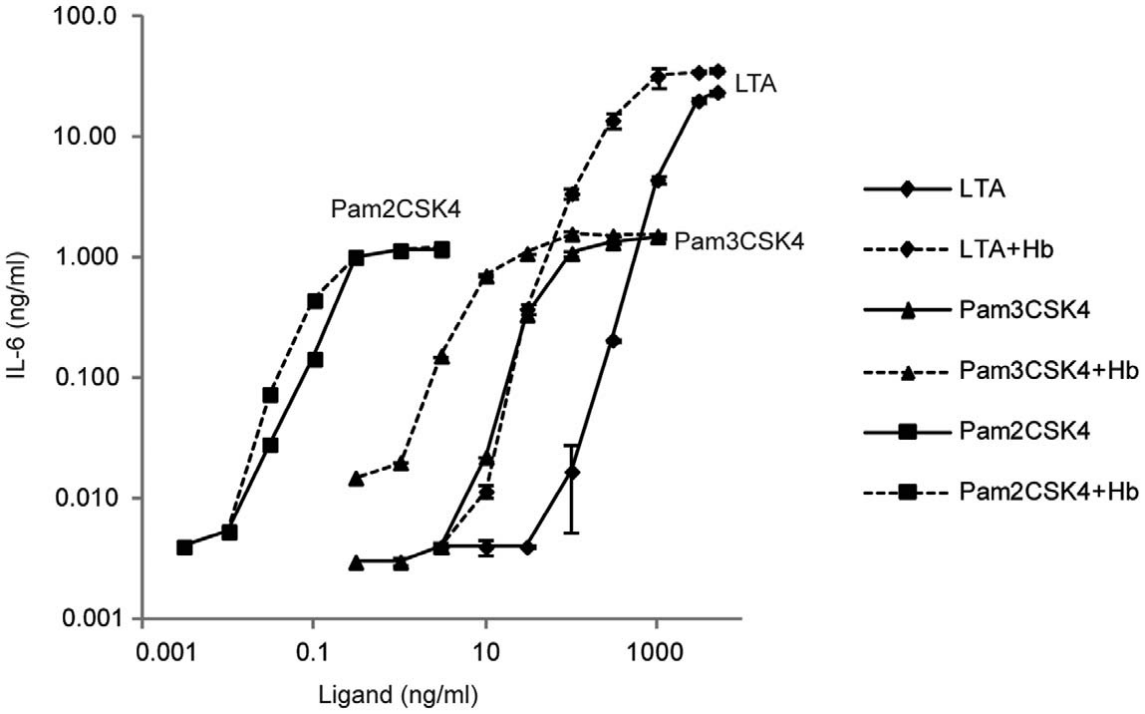
Macrophage dose response curves for IL-6 secretion induced by TLR2 ligands with and without Hb. HeNC2 cells were stimulated with various amounts of LTA (diamonds), Pam3CSK4 (triangles) or Pam2CSK4 (squares) with or without Hb (50 µg/ml, dashed lines or solid lines, respectively) for 18 hours at 37°C. Levels of IL-6 in the supernatants were analyzed by ELISA. Data are plotted on a log-log scale. These results denote mean +/− standard error (n = 3) and are representative of three independent experiments.

We have previously shown that activating either murine peritoneal macrophages or human blood cells with LTA plus Hb significantly enhanced the secretion of IL-6 when compared to activating with LTA alone, even though incubation with Hb alone resulted in no detectable secretion of IL-6 [25]. To determine whether other TLR2 ligands evoke increased macrophage cytokine secretion in the presence of Hb, we incubated HeNC2 cells with Pam2CSK4, Pam3CSK4 or LTA, with or without 50 µg/ml Hb. There was an increase in IL-6 secretion for all three ligands when Hb was added (Fig. 1). Incubation with Hb alone, as shown previously, did not result in any detectable secretion of IL-6 (data not shown). The greatest differences in IL-6 levels were 200-, 40-, and 3-fold for activation by LTA, Pam3CSK4 and Pam2CSK4, respectively, and these levels occurred at low concentrations of ligand for LTA and Pam3CSK4. In each case, the enhanced IL-6 secretion due to addition of Hb was minimized or lost entirely when ligand concentrations were sufficiently high. Interestingly, macrophages were more sensitive to both lipopeptides, on either a weight or molar basis, but the maximum response and synergy with Hb were greater when cells were stimulated with LTA.

### Macrophage Transcriptome following Stimulation with LTA, Hb or LTA Plus Hb

To begin to determine the molecular basis of this enhanced cytokine production, we examined the transcriptome of HeNC2 cells activated by LTA, Hb, LTA plus Hb or cells maintained in medium alone. RNA from three separate biological replicates was obtained 6 hours after initiation of stimulation. This time point was chosen based on a previous time-course showing that by 8 hours one could detect significant differences in the response of HeNC2 cells to LTA plus Hb as compared to LTA for both early (e.g. TNF-α) and late response (e.g. IL-6) proteins [26].

**Figure 2:**
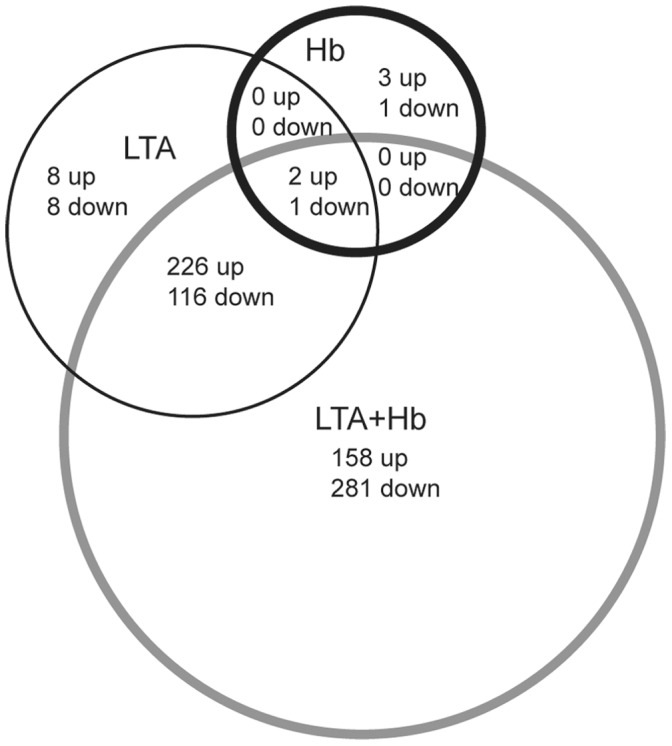
Venn diagram showing the number of differentially activated genes. HeNC2 cells were activated with LTA, Hb, LTA plus Hb or maintained in medium alone and RNA was obtained 6 hours post-treatment. Differentially expressed genes are defined as those with a GCOS-generated detection value >0.05 for at least the experimental value, a t test p value of <0.05 and a 1.5-fold or greater change between the experimental value and the medium control. These differentially expressed genes are listed in Supplemental Tables 2–4.

Microarray analysis revealed that a total of 784 genes were affected by LTA plus Hb activation. 386 of these genes were up-regulated, while 398 genes were down-regulated (Fig. 2, Supplemental Table 2). Of these 784 genes, 342 were also affected by LTA activation (Fig. 2, Supplemental Table 3). Surprisingly, only 7 genes responded to Hb alone at the 6-hour time point and, of these genes, 3 were also up- or down-regulated by LTA plus Hb, including the gene encoding TNF-α (Fig. 2, Supplemental Table 4). Approximately 90% of the genes regulated by activation of HeNC2 cells with LTA plus Hb are also known to be regulated by Pam3CSK4 and/or LPS [35], [36].

To categorize the sets of genes that were expressed only when HeNC2 cells were stimulated with LTA plus Hb according to their function, we performed functional annotation analysis using the DAVID Bioinformatics Resources [37], [38]. We found 12 Gene Ontology Biological Processes that were enriched in genes up-regulated only after stimulation with LTA plus Hb and 11 that were enriched in down-regulated genes. The categories of down-regulated genes were mainly those involving DNA replication, although there were also intracellular signal cascade genes and genes involving apoptosis (Supplemental Table 5). Certain intracellular signaling cascade genes and genes involving apoptosis were also present among the up-regulated genes. In addition, there were 11 genes with transcription factor activity, including IRF5, IRF7, CREB5, NF-κB2 and STAT2 (Supplemental Table 5).

The array data were also examined to define genes that showed at least 2.5-fold greater up-regulation when data from macrophages that were activated by LTA plus Hb were compared to data from macrophages that were activated by LTA alone (Table 1). The majority of these genes are chemokines, cytokines, receptors and IFN-induced genes.

### Temporal Expression Patterns of Cytokines and Chemokines Preferentially Up-Regulated by LTA Plus Hb

**Figure 3:**
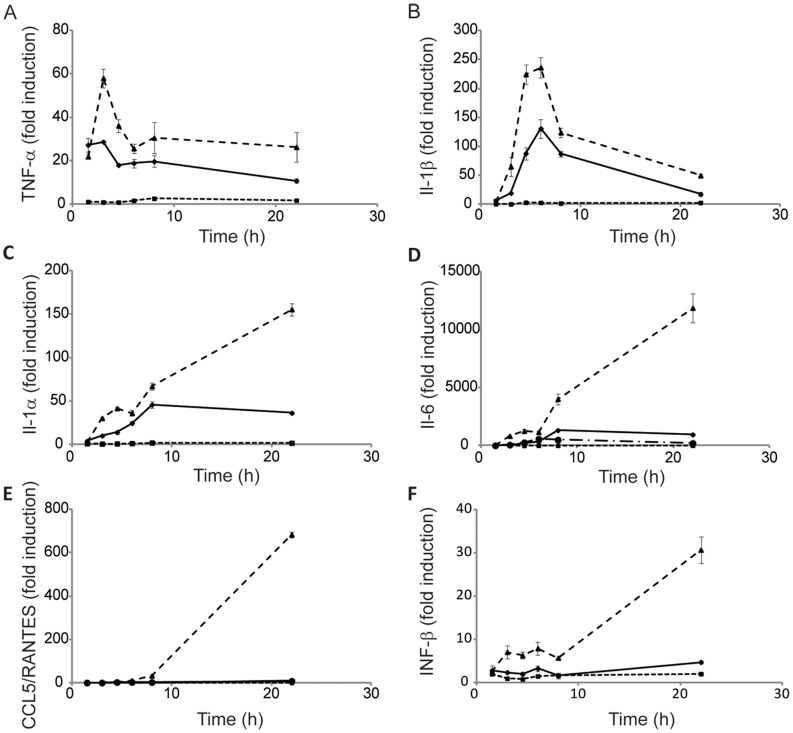
qRT-PCR analysis of macrophage gene expression. Tlr4^+^/_+_ HeNC2 cells were stimulated with either LTA (2 µg/ml, diamonds, solid line), Hb (50 µg/ml, squares, short dashes), LTA+Hb (2 µg/ml and 50 µg/ml, respectively, triangles, long dashes) or maintained in medium for the indicated time period and then analyzed by qRT-PCR for TNF-α (**A**), IL-1β (**B**), IL-1α (**C**), IL-6 (**D**), CCL5/RANTES (**E**) or IFN-β (**F**) mRNA levels compared to control. Tlr4^d^/_d_ GG2EE cells were stimulated with LTA+Hb (2 µg/ml and 50 µg/ml, respectively; circles, dashes and dots) and assayed for IL-6 (D) or CCL5/RANTES (E). These results denote mean +/− standard error (n = 3) and are representative of three independent experiments.

Since only one time point was analyzed by microarray, qRT-PCR was used to evaluate the temporal expression patterns of some of the major cytokines and chemokines that the microarray results showed to be preferentially up-regulated in HeNC2 cells activated with LTA plus Hb compared to LTA alone. In addition to validating the array data, qRT-PCR analysis provided more insight into the potentiation of LTA-stimulated cytokine and chemokine expression by Hb. The 5 genes that we report here represent two different response patterns (Fig. 3). TNF-α and IL-1β mRNA levels both increase quickly after the addition either of LTA or LTA plus Hb and peak between 3 and 6 hours post-activation (Fig. 3A, B). A similar pattern was observed for IL-1α and IL-6 after stimulation of HeNC2 cells with LTA alone, although peak accumulation occurred at later times (Fig. 3C, D). CCL5/RANTES mRNA accumulation after stimulation by LTA was essentially at control levels (Fig. 3E). However, a very different pattern of expression was seen after stimulating HeNC2 cells with LTA plus Hb. Instead of a gradual decline following the initial burst, IL-1α, IL-6 and CCL5/RANTES mRNA levels increased substantially between 8 and 22 hours post-activation.

To determine whether this large increase in cytokine and chemokine mRNA after 8 hours of activation by LTA plus Hb was TLR4-dependent, mRNA levels from wild-type HeNC2 cells were compared to those from TLR4 mutant GG2EE cells [33]. The large increase in IL-6 and CCL5/RANTES mRNA after LTA plus Hb activation that was observed between 6 and 22 hours in wild-type cells was absent when mRNA from the GG2EE cell line was assayed by qRT-PCR (Fig. 3D,E), showing that the response was TLR4-dependent. In addition, even earlier time points after activation with LTA plus Hb showed a decrease of IL-6 mRNA in TLR4-deficient cells.

### NF-κB Activation Kinetics

**Figure 4:**
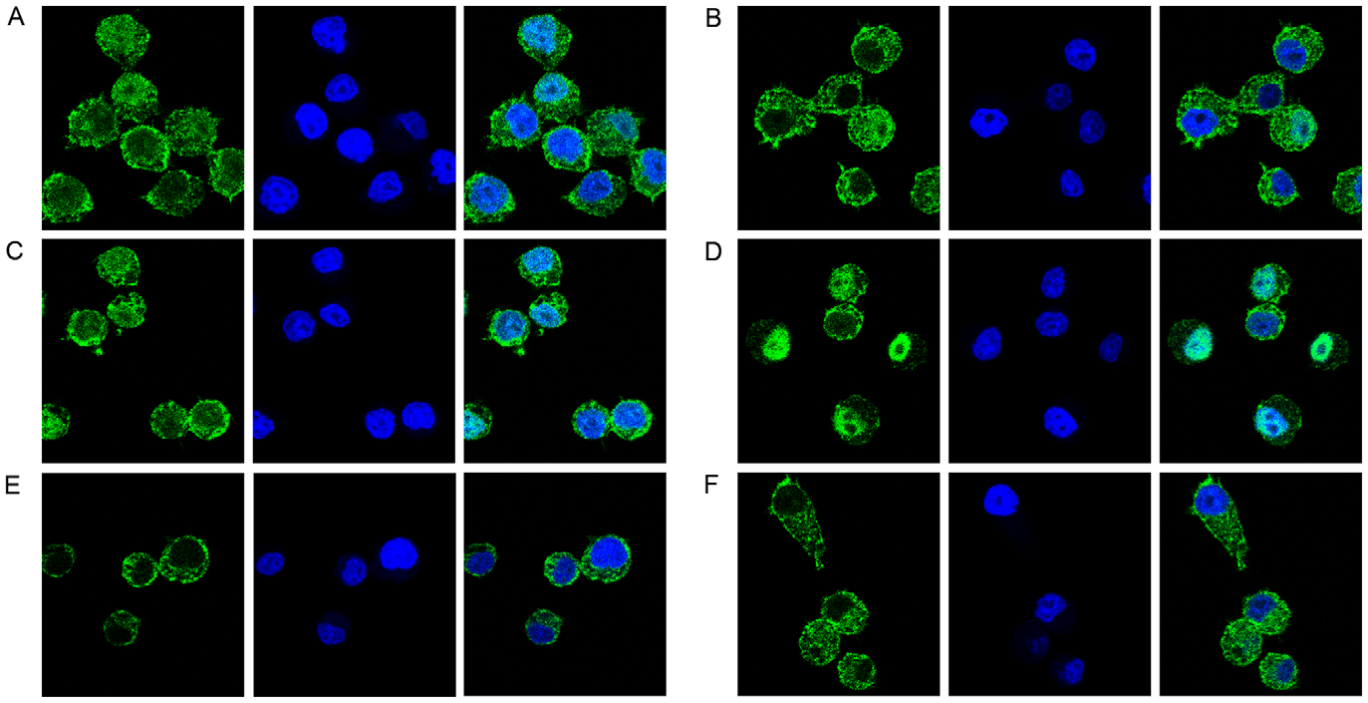
Cellular localization of NF-κB p65 in HeNC2 cells. HeNC2 cells were stimulated with Pam2CSK4 (100 ng/ml, panel A), Pam2CSK4 plus Hb (100 ng/ml and 50 µg/ml, respectively, panel B), LTA (1 µg/ml, panel C), LTA plus Hb (1 µg/ml and 50 µg/ml, respectively, panel D), Hb (50 µg/ml, panel E) or maintained in medium (panel F) for 4 hours. Cells were then fixed, permeabilized, blocked and incubated with NF-κB p65 overnight at 4°C. After washing, cells were incubated with anti-rabbit IgG conjugated to Alexa Fluor-488 (green). Cells were also stained with SYTOX Orange (blue) to visualize the nuclei and then analyzed by confocal microscopy.

qRT-PCR results showed that mRNA levels of several genes preferentially up-regulated by LTA plus Hb compared to LTA alone continued to increase later in the activation process. To determine if LTA plus Hb might induce a unique NF-κB response when compared to other TLR2 ligands, we examined the cellular localization of NF-κB. In unstimulated cells, NF-κB is sequestered in the macrophage cytoplasm by interaction with its inhibitor IκBα [39]. Stimulus-induced degradation of IκBα releases NF-κB and allows its rapid accumulation in the nucleus where it binds to κB DNA sequences and activates a set of genes [40], [41]. Later, newly synthesized IκBα dissociates NF-κB from the DNA and allows its nuclear export, thus determining the duration of the NF-κB response [42]. We examined the nuclear localization of NF-κB at later time points by immunofluorescence. Figure 4 shows that at 4 hours post-stimulation, the only set of cells in which NF-κB remains primarily localized to the nucleus are those stimulated with LTA plus Hb (panel D).

**Figure 5:**
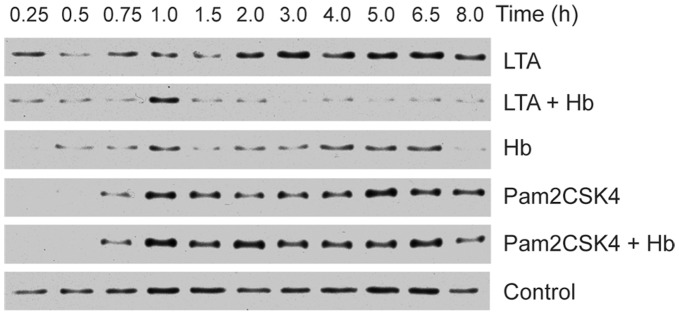
Degradation of IκBα during macrophage activation. HeNC2 cells were stimulated with LTA (1 µg/ml), LTA plus Hb (1 µg/ml and 50 µg/ml, respectively), Hb (50 µg/ml), Pam2CSK4 (100 ng/ml), Pam2CSK4 plus Hb (100 ng/ml and 50 µg/ml, respectively) or maintained in medium for the indicated times. Cell extracts were electrophoresed on SDS-PAGE gels, transferred to nitrocellulose and incubated with anti-IκBα. Similar results were obtained from each of two independent experiments.

To determine if the extended nuclear localization of NF-κB was reflected in an absence of its inhibitor, we examined the level of IκBα in these cells. It had been shown previously that activation of immortalized or primary macrophages with Pam3CSK4 led to a rapid degradation of IκBα, which became undetectable within 15 minutes post-stimulation but was resynthesized to pre-induced levels by 1 hour [43]. Stimulation of HeNC2 cells with Pam2CSK4 caused a rapid and complete loss of IκBα by 15 minutes and return to pre-induced levels by 1 hour as predicted, and stimulation with Pam2CSK4 plus Hb exhibited a similar profile (Fig. 5). In comparison, degradation of IκBα following stimulation by LTA was delayed and did not return to pre-stimulatory levels until around 2 hours post-stimulation. However, cells stimulated with LTA plus Hb showed a substantial difference in IκBα degradation kinetics. IκBα was partially degraded by 15 minutes and remained at low levels throughout the 8-hour time period, except for a brief return to pre-stimulation levels at the 1 hour time point. To our knowledge, such a prolonged absence of cytoplasmic IκBα has not been observed after stimulation with any other TLR2 ligand, including the triacylated lipopeptide, Pam3CSK4 (TLR1/TLR2) or the diacylated lipopeptides, Pam2CSK4 (TLR2/TLR6), S-FSL-1 (TLR2/TLR6) and R-FSL1 (TLR2/TLR6/CD-36) [44]. Hb activation also resulted in IκBα levels that were lower than pre-stimulatory levels at some time points. However at 4 hours post-stimulation, the time point shown in figure 4, the level of IκBα is high and that is reflected by the cytoplasmic localization of NFκB (panel E). These data show that LTA plus Hb stimulation results in the prolonged nuclear localization of the transcription factor NFκB and the absence of its inhibitor, IκBα. This unique continuation of NFκB nuclear localization may have led to the extended synthesis of NF-κB-regulated genes observed in Figure 3.

### An Endocytosis-dependent Pathway is Required for Maximal IL-6 Secretion

**Figure 6:**
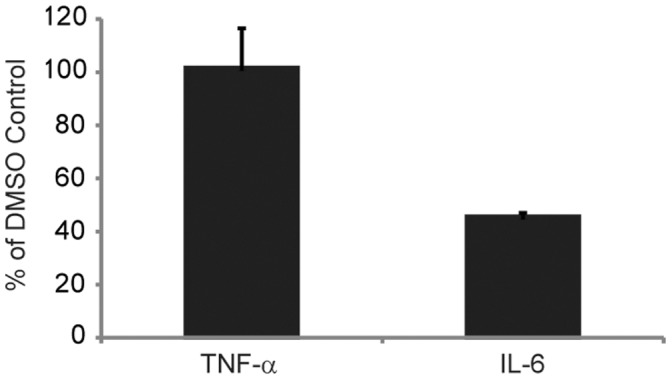
Effect of Dynasore® on macrophage TNF-α and IL-6 secretion induced by LTA plus Hb. HeNC2 cells were preincubated with either 40 µM Dynasore® or an equivalent amount of the solvent, DMSO, for 30 min and then stimulated with LTA plus Hb (2 µg/ml and 50 µg/ml, respectively) for 6 hours. TNF-α and IL-6 concentrations were analyzed by ELISA. These results denote mean +/− standard error (n = 3) and are representative of three independent experiments.

Since the maximal secretion of IL-6 after stimulation of macrophages by LTA plus Hb is TLR4-dependent [26], we determined whether cytokine and chemokine secretion involved an endocytic signaling pathway, such as the TRIF/TRAM-dependent pathway. We examined activation of HeNC2 cells in the presence of Dynasore®, a chemical inhibitor of caveolin- and clathrin-dependent endocytic pathways by virtue of its inhibition of dynamin [45]. Kegan et al. have demonstrated that dynamin controls TLR4 endocytosis and that activation of macrophages in the presence of Dynasore® inhibits the TRIF/TRAM pathway [13]. HeNC2 cells were pre-treated with 40 µM Dynasore® and then stimulated with LTA plus Hb for 6 h. Secretion of the MyD88-dependent, TRIF-independent gene product, TNF-α, and the MyD88-dependent, TRIF-dependent gene product, IL-6, were assayed [46]–[48]. Dynasore® treatment did not affect the secretion of TNF-α, but it greatly reduced the level of IL-6 (Fig. 6). This demonstrates that a significant proportion of the secretion of IL-6 is dependent upon an endocytic pathway in LTA plus Hb activated HeNC2 cells.

### Macrophage Activation by LTA Plus Hb Results in IFN-β Expression

The endocytic TRIF/TRAM-dependent pathway leads to the expression of type 1 interferons. Since 6 of the 27 genes that were most highly up-regulated by LTA plus Hb when compared to LTA alone were interferon (IFN)-regulated, qRT-PCR was used to evaluate expression of the IFN-β gene during LTA plus Hb activation. IFN-β mRNA stimulated by LTA plus Hb exhibited an early peak around 6 hours (Fig. 3F). Between 8 and 22 hours, message accumulation increased further, similar to the pattern seen for IL-6, IL-1α and CCL5 mRNA. This provides additional evidence that cells stimulated by LTA plus Hb activate an endocytic pathway that leads to the expression of the IFN-β gene, most likely via the TRIF/TRAM-dependent endocytic pathway.

### Hb Stimulates TLR4-dependent TNF-α Secretion

**Figure 7:**
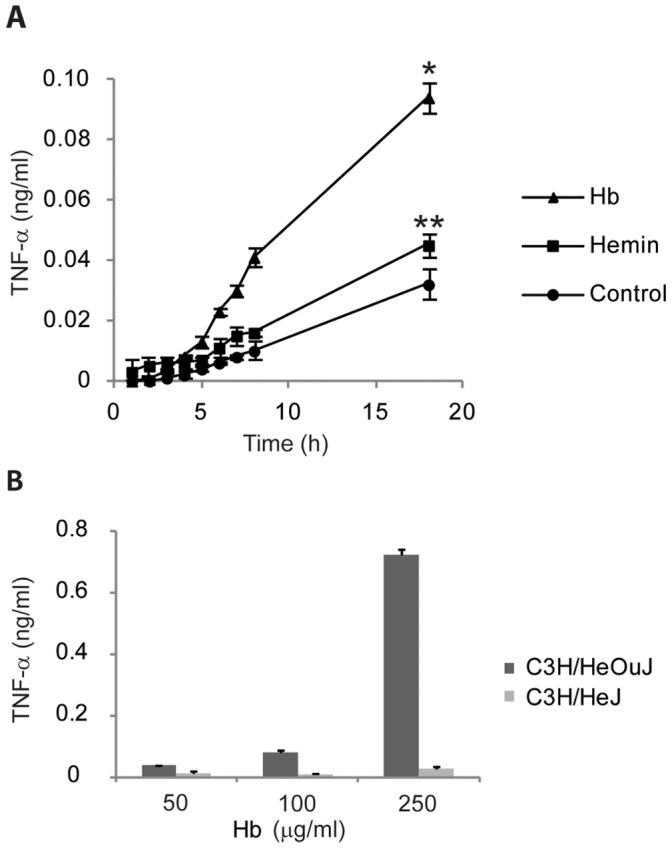
Macrophage TNF-α secretion induced by Hb. (**A**) HeNC2 cells were incubated with Hb (50 µg/ml), hemin (6.5 µg/ml) or maintained in medium for the indicated times and TNF-α concentrations were determined by ELISA. (**B**) Bone marrow macrophages from C3H/HeOuJ (TLR4^+^/_+_) and C3H/HeJ (TLR4 ^d^/_d_) mice were stimulated with various amounts of Hb for 18 hours. TNF-α concentrations were determined by ELISA. *P<0.02 Hb activation compared to control at 18 hours. **P>0.05 Hemin activation compared to control at 18 hours. These results denote mean +/− standard error (n = 3) and are representative of three independent experiments.

When Hb is added to LTA, maximal secretion of IL-6 from macrophages requires TLR4 [26]. We previously ruled out Hb as a TLR4 ligand, since incubation of macrophages with Hb alone did not result in detectable secretion of IL-6. In light of the microarray and qRT-PCR analyses that showed the presence of TNF-α mRNA above control levels when cells were stimulated with Hb alone, we revisited the idea that activation of HeNC2 cells with Hb alone might result in TNF-α secretion. Indeed, Hb did induce low but statistically significant levels of TNF-α secretion from HeNC2 cells (Fig. 7A). Furthermore, this stimulation required more than just the hemin group, since hemin in roughly 15-fold molar excess compared to the Hb used showed no ability to elicit a statistically significant amount of TNF-α secretion (Fig. 7A), nor did it potentiate the macrophage response to LTA, yielding TNF-α levels that were essentially identical to those elicited by LTA alone (data not shown).

To determine if Hb stimulation was TLR4-dependent, bone marrow-derived macrophages (BMM) from wild-type C3H/HeOuJ (Tlr4^+^/_+_) and mutant C3H/HeJ (Tlr4^d^/_d_) (4) mice were stimulated with various concentrations of Hb. BMM derived from C3H/HeJ mice released far less TNF-α than BMM from C3H/HeOuJ mice, demonstrating that Hb stimulation of macrophages leading to TNF-α secretion is primarily TLR4-dependent (Fig. 7B).

### High Mobility Group Box 1 Protein (HMGB1) is Released during Hb Activation of Macrophages

**Figure 8:**
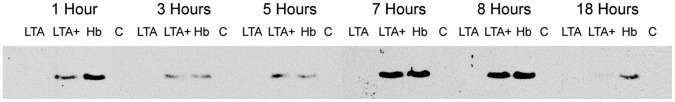
Secretion of HMGB1 by macrophages activated by LTA, LTA+Hb or Hb alone. HeNC2 cells were stimulated with LTA (LTA, 1 µg/ml), LTA+Hb (LTA+, 1 µg/ml and 50 µg/ml respectively), Hb alone (Hb, 50 µg/ml) or maintained in medium (C) for the indicated times. Supernatants from these incubations were electrophoresed on SDS-PAGE gels, transferred to nitrocellulose and incubated with anti-HMGB1. Similar results were obtained from each of two independent experiments.

**Figure 9:**
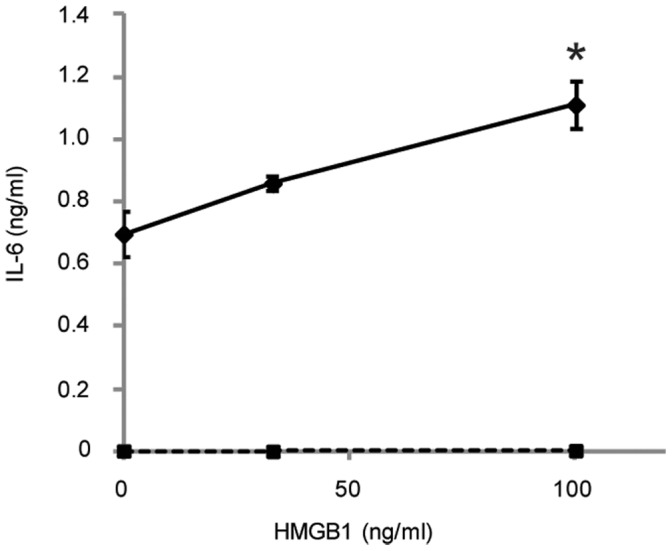
Effect of purified recombinant HMGB1 on IL-6 secretion by macrophages. HeNC2 cells were stimulated with various amount of HMGB1 with LTA (solid line, 1 µg/ml)) or without (dashed line). IL-6 concentrations were determined by ELISA. *P<0.02 Activation with LTA plus 100 ng/ml HMGB1 compared to activation with LTA alone. These results denote mean +/− standard error (n = 3) and are representative of three independent experiments.

Hb may be acting as a TLR4 ligand in this system or it may stimulate the secretion of another TLR4 ligand. HMGB1, an alarmin and suspected TLR4 agonist [49], [50], has been shown to be secreted after stimulation of TLR4 in macrophages [51]. The supernatants of HeNC2 cells activated by LTA, LTA plus Hb, or Hb were analyzed for the presence of HMGB1 by Western blot. HMGB1 was observed in the medium by 1 hour whether cells were stimulated by LTA plus Hb or Hb alone, but not after stimulation with LTA alone (Fig. 8). HMGB1 rose to peak levels at 7–8 hours. To determine more directly whether HMGB1 could activate HeNC2 cells, we measured levels of IL-6 and TNF-α in cells treated with purified recombinant HMGB1. Recombinant HMGB1 alone did not activate HeNC2 cells to secrete IL-6 (Fig. 9) or TNF-α (data not shown). Using Western blot analysis of known amounts of recombinant HMBG1, we estimated the level of HMGB1 in the supernatant of HeNC2 cells activated with LTA plus Hb at 8 hours to be approximately 100 ng/ml (data not shown). We activated HeNC2 cells with HMGB1 and LTA to determine if any increase in cytokine secretion would result and showed that adding 100 ng/ml recombinant HMGB1 to LTA increased the amount of IL-6 secretion approximately 1.5 fold after 22 hours (Fig. 9).

## Discussion

It is clear that free Hb and a growing number of bacterial molecules can synergize during macrophage activation to result in enhanced innate immune responses [52]–[57]. This observation is biologically important because even though the body has highly efficient systems for scavenging Hb and heme under physiological conditions, there are a myriad of pathological states in which these systems can be overwhelmed, exposing the tissues to free Hb [58]–[60]. In addition, a number of Gram-positive pathogens produce hemolysins that could also increase the concentration of free Hb [29]. When Hb, which does not induce the secretion of IL-6 on its own, was added to various TLR2 ligands, including LTA, the level of secreted IL-6 was increased and this synergy was maximal at the lowest levels of the TLR2 ligands. This finding suggests that in order for the interactions between macrophages, the bacterial products, and Hb to play an important role in innate immune responses in specific microenvironments, the concentrations of these components do not have to be high. These microenvironments may include areas where there is minor capillary damage and leakage in conjunction with bacterial components, such as in arterial plaques [27] and in periodontal tissues [28]. Hb has also been shown to synergize with the potent TLR4 ligand, LPS, and significantly enhance its biological activity [61]–[64], leading to increased mortality from LPS in a mouse model [65]. Ligands for TLR3, TLR7 and TLR9 also show enhanced macrophage activation in the presence of Hb [52].

The microarray data defined the group of genes that was most highly up-regulated as a result of synergy between LTA and Hb when compared to LTA alone. The majority of this group of 27 genes were cytokines, chemokines, receptors, and IFN-regulated genes. This group contains the chemokines CCL2/MCP1, CCL5/RANTES, and CXCL10/IP-10, which are known to be secreted within human atherosclerotic lesions [66] as well as CCRL2, a chemokine receptor that has been reported to bind and be activated by CCL2, CCL5 and CCL7 [67].

Data from qRT-PCR experiments have begun to define the mechanism by which Hb synergizes with LTA to amplify the immune response. When LTA plus Hb was used to activate macrophages, IL-6 cytokine levels continued to rise at least through 32 hours following activation, and this increase was TLR4-dependent [26]. Of the 5 cytokines or chemokines that were analyzed by qRT-PCR, 3 of them, including IL-6, showed very high levels of mRNA that continued to increase between 8 and 22 hours after activation with LTA plus Hb, and, again, this increase was TLR-4 dependent. The elevated levels of these cytokine and chemokine mRNAs at these late time points is unique and not seen when macrophages were stimulated with LPS or Pam3CSK4 [35], [36]. We do not know at present whether the enhanced long-term accumulation of these mRNA species is due to extended transcriptional activation or post-transcriptional regulation. However we have observed that the nuclear localization of NF-kB and absence of IκBα, the inhibitor of NF-κB, is uniquely prolonged when cells are stimulated by LTA plus Hb. This suggests that genes controlled by the transcription factor, NF-κB, may continue to be synthesized over a longer period of time post-stimulation. This is in sharp contrast to our data showing that after activation with LTA or Pam2CSK4, IκBα levels returned to pre-activation levels by 2 and 1 hour, respectively and NF-kB was localized primarily to the cytoplasm by 4 hours post-activation. In addition to transcriptional control, maintenance of high mRNA levels long after activation may be influenced by post-transcriptional regulation, such as down-regulation of some microRNAs which have been shown to decrease the stability of mRNA species. For example, MicroRNA-27b has been shown to have a role in LPS-induced expression levels of TNF-α and IL-6 by modulating mRNA stability [68], [69].

Following activation of macrophages with LTA plus Hb, we detected IFN-β mRNA at levels above control. In addition, an endocytic pathway and the TLR4 receptor are required for maximum secretion of IL-6. These findings imply that the endocytic TRIF/TRAF-dependent pathway is probably utilized during activation of macrophages by LTA plus Hb. The activation of the TRIF/TRAF-dependent pathway may account for the increased level of some cytokine and chemokine mRNA at later time periods after activation, as well as the expression of IFN-regulated genes since this pathway results in a second activation of NF-κB along with the phosphorylation of the IRF3 transcription factor. Activation of IRF3 results in dimerization and the translocation of IRF3 to the nucleus and the expression of type 1 interferons (e.g. interferon beta) and interferon-inducible genes.

Hb may be the TLR4 ligand that activates this pathway. Hb alone can activate macrophages to secrete modest amounts of TNF-α, and this TNF-αsecretion is TLR4-dependent. However, activation of macrophages by Hb does not result in the expression of the same set of genes or secretion of the same set of gene products that a high concentration of LPS, a well-studied TLR4 ligand, would elicit. For example, after 6 hours of Hb activation the microarray shows that the only cytokine or chemokine mRNA transcribed above control levels was TNF-αmRNA. In addition, secreted TNF-α, but not IL-6, was detected after activation of macrophages with Hb. This result is very similar to that of Figueiredo et al [70] who showed that hemin at very high concentrations could induce TLR4-dependent secretion of TNF-αfrom macrophages, but not IL-6. It remains possible that an alternative signal transduction response other than that observed when macrophages are stimulated with high levels of LPS could be obtained with different ligands or concentrations of ligands. Supporting this proposal is the observation that activation of macrophages with low concentrations of LPS (50–100 pg/ml) results in a unique TLR4 macrophage response in which C/EBP is selectively activated and other nuclear repressors are removed, resulting in a modest expression of cytokines and chemokines without the activation of NF-κB [71].

At this point, we do not know whether Hb itself or another molecule elicited from the macrophages by Hb is the TLR4 ligand. We found that HMGB1, a molecule proposed to stimulate cells through multiple receptors including TLR4 [72]–[74], was released from cells incubated with Hb alone. HMGB1, which normally acts intracellularly as a nuclear non-histone DNA-binding protein [75], has been proposed to act as a cytokine that mediates a response to infection and inflammation when it is released actively from intact cells or passively from necrotic cells [76]. Activation of the macrophage through the TLR4 receptor can induce the release of HMGB1 through the IFN-β-mediated JAK/STAT pathway [51]. Whether HMGB1 has cytokine functions on its own or binds to and synergizes with other PAMPs is still unclear [77]. Our data showed that highly purified, recombinant HMGB1 alone did not activate HeNC2 cells to release detectable amounts of TNF-α or IL-6, but it is not known whether the activity of this recombinant protein is equivalent to native protein [78], [79]. Recombinant HMGB1 in combination with LTA did result in higher IL-6 secretion than with LTA alone, demonstrating synergy between HMGB1 and LTA, as has been reported between HMGB1 and LPS [80], [81]. Thus, it is possible that the synergy between HMGB1 and LTA may account for a portion of the increased secretion of cytokines and chemokines observed when macrophages are activated with LTA plus Hb.

## Supporting Information

Table S1 **List of primers used for quantitative RT-PCR of cytokines and chemokines.**
(PDF)

Table S2 **Microarray results of transcripts that showed a greater than 1.5-fold difference in expression between LTA plus Hb and control.**
(XLSX)

Table S3 **Microarray results of transcripts that showed a greater than 1.5-fold difference in expression between LTA and control.**
(XLSX)

Table S4 **Microarray results of transcripts that showed greater than 1.5-fold difference in expression between Hb and control.**
(XLSX)

Table S5 **Enrichment of Gene Ontology Terms for genes expressed only when macrophages were stimulated by LTA plus Hb.**
(XLSX)
