# Supplementary material for: Pathways Involved in the Synergistic Activation of Macrophages by Lipoteichoic Acid and Hemoglobin
Source: PLoS One. 2012 Oct 10;7(10):e47333. doi: 10.1371/journal.pone.0047333 (PMC3468568; doi:10.1371/journal.pone.0047333)
Supplement: Table S1 — List of primers used for quantitative RT-PCR of cytokines and chemokines. (PDF) [file pone.0047333.s001.pdf]

Supplemental Table 1
Primers used for qRT-PCR of cytokines and chemokines

GENE	SENSE PRIMER	ANTISENSE PRIMER
IL-1α	5’-GAGCGCTCACGAACAGTTG-3’	5’-TTGGTTAAATGACCTGCAACA-3’
IL-1β	5’-TCTTCTTTGGGTATTGCTTGG-3’	5’-TGTAATGAAAGACGGCACACC-3’
IL-6	5’-TTCATGTACTCCAGGTAGCTATGG-3’	5’-TGATGGATGCTACCAAACTGG-3’
IFN-β	5’-CATTTCCGAATGTTCGTCCT-3’	5’-CACAGCCCTCTCCATCAACTA-3’
RANTES	5’-GAGTGGTGTCCGAGCCATA-3’	5’-TGCAGAGGACTCTGAGACAGC-3’
TNF-α	5’-GGTCGGGCCATAGAACTGA-3’	5’-TCTTCTCATTCCTGCTTGTGG-3’
HGPRT	5’-CCTGGTTCATCATCGCTAATC-3’	5’-TCCTCCTCAGACCGCTTTT-3’
